# Understanding the role of international flight networks in disease spread: spatial epidemic prevention zones and hierarchical disease control policies

**DOI:** 10.1186/s12942-026-00471-9

**Published:** 2026-05-18

**Authors:** Shan-Yin Yang, Wei Chien Benny Chin, Tzai-Hung Wen, Pei-Fen Kuo, Chun-Hsiang Chan

**Affiliations:** 1 Graduate Institute of Electronics Engineering, National Taiwan University, Taipei City, Taiwan; 2 Department of Geography, National University of Singapore, Queenstown, Singapore; 3 Department of Geography, National Taiwan University, Taipei City, Taiwan; 4 Department of Geomatics, National Cheng Kung University, Tainan City, Taiwan; 5 Department of Geography, National Taiwan Normal University, Taipei City, Taiwan

**Keywords:** COVID-19, Global country network, Spatial epidemic prevention zone, Hierarchical disease control policy

## Abstract

This study analyzed the January 2020 international flight network, i.e., during the early global spread of COVID-19, to examine how global air connectivity shaped the timing of first imported cases and to derive policy-relevant spatial risk groupings. Infomap was used to delineate spatial epidemic prevention zones (SEPZs), and network indicators, including PageRank, betweenness centrality, the clustering coefficient, and hyperlink-induced topic search (HITS), were used to characterize the international passenger arrival probability, transfer-related connectivity, connection density, and spatial potential transmission. Passenger arrival probability and connection density were associated with a shorter time to arrival (T2A) of the first imported COVID-19 case, whereas a greater geographical distance was associated with a longer T2A. In contrast, macroscale socioeconomic indicators showed limited explanatory power in the early disease transmission. Building on the SEPZs and spatial transmission potential, we propose a hierarchical disease control framework as a decision-support tool for early-stage cross-border risk assessment and scenario planning.

**Supplementary Information:**

The online version contains supplementary material available at 10.1186/s12942-026-00471-9.

## Introduction

In today’s interconnected world, international air travel forms a dynamic relational space that fosters global exchange but also accelerates the cross-border spread of infectious diseases [1]. During the early phase of a global outbreak, air networks are analytically more informative than geographic proximity alone because they capture directed passenger flows, transfer connectivity, and functional proximity between countries. The early global spread of COVID-19 illustrates this mechanism clearly: within a short period, the virus was seeded across multiple continents through highly connected air routes [2–5]. These patterns underscore why flight frequency, passenger volume, and network position should be incorporated into spatially explicit models for pandemic preparedness [6, 7].

Countries adopted layered control strategies that combined border measures with individual-level protection to mitigate COVID-19 transmission. Governments applied temperature screening and travel restrictions, testing, vaccination requirements, and quarantine policies to reduce imported infections and downstream local spread [8–14]. However, these interventions differed substantially in timeliness, stringency, and effectiveness, highlighting the need for analytically grounded frameworks that can support more differentiated and network-aware responses.

This policy landscape reveals a core trade-off: broad restrictions may reduce importation risk, but they can also disrupt mobility, trade, tourism, and routine social life [15–18]. The practical challenge is therefore not simply whether to intervene, but how to calibrate interventions so that they reflect heterogeneous cross-border transmission risk rather than treating all origins and destinations as epidemiologically equivalent.

Existing zoning studies can be broadly grouped into two conceptual categories. First, adjacency-based frameworks delineate control areas primarily through spatial proximity or administrative contiguity [19–21]. Second, mobility-informed frameworks delineate functionally connected zones using regular travel, commuting, amenity-sharing, or public transportation flows [19, 20, 22–24]. Although these approaches have improved local disease control planning, most were developed for intra-urban or national settings, leaving a major gap in international-scale zoning and intervention design.

That gap became especially visible during the COVID-19 pandemic. International air travel is a primary conduit of rapid cross-border transmission, and the position of countries within the global flight network strongly shapes early importation timing [25–32]. Yet a cohesive framework that translates global network structure into spatially differentiated and policy-relevant prevention strategies remains underdeveloped.

In addition to zone delineation zones, network analysis can clarify identifying the roles of countries in the disease-spreading process [33, 34]. Simple inflow or outflow statistics describe direct traffic magnitude, but they do not fully capture whether a country functions as a receiver, bridge, or spreader within the broader directed network. Previous studies have demonstrated the network-based indicators can improve the understanding of importation timing, bridge-like connectivity, and spatial vulnerability in transportation systems [35–38]. These findings motivate the use of recursive and topology-aware metrics in the international flight context.

Accordingly, the present study concentrates on the early global spread of COVID-19 and uses international flight data to examine how network structure, spatial clustering, and temporal importation patterns can be integrated into an operational analytical framework for epidemic preparedness.

This study introduces a computational framework that integrates a complex spatial network analysis to evaluate country-level vulnerability to early cross-border disease transmission. Using the January 2020 international air travel network and first imported COVID-19 case data, we delineate global spatial epidemic prevention zones (SEPZs), assess how network properties shape time to arrival, identify temporal infection clusters, and derive a spatial transmission potential (STP) for risk stratification. By adopting countries and regions as the primary decision-making unit, the model is designed to support spatially differentiated, network-aware scenario planning rather than a universal prescriptive policy.

## Materials and methods

The specific aims of this study are threefold: (1) to reveal the roles of global country network properties in disease-spreading propagation; (2) to delineate spatial epidemic prevention zones (SEPZs) and validate the relationship between SEPZs and disease-spreading propagation; and (3) to establish a hierarchical disease control policy based on global country network properties.

### Materials

Four datasets were collected and utilized in this study: (1) global country network data, (2) first imported COVID-19 case data, (3) socioeconomic status data, and (4) international travel restriction data.

Collected from the Official Aviation Guide (OAG), the global country network dataset includes air transportation data from January 2020 for 271 countries and regions, totaling 1,837,787 records [39]. The dataset contains the details of routes, including departure locations (airport IATA code and country name), transfer locations, arrival locations, and the total estimated number of passengers. Prepandemic (January 2020) data were used to capture normal, undisturbed air transportation patterns.

**Fig. 1:**
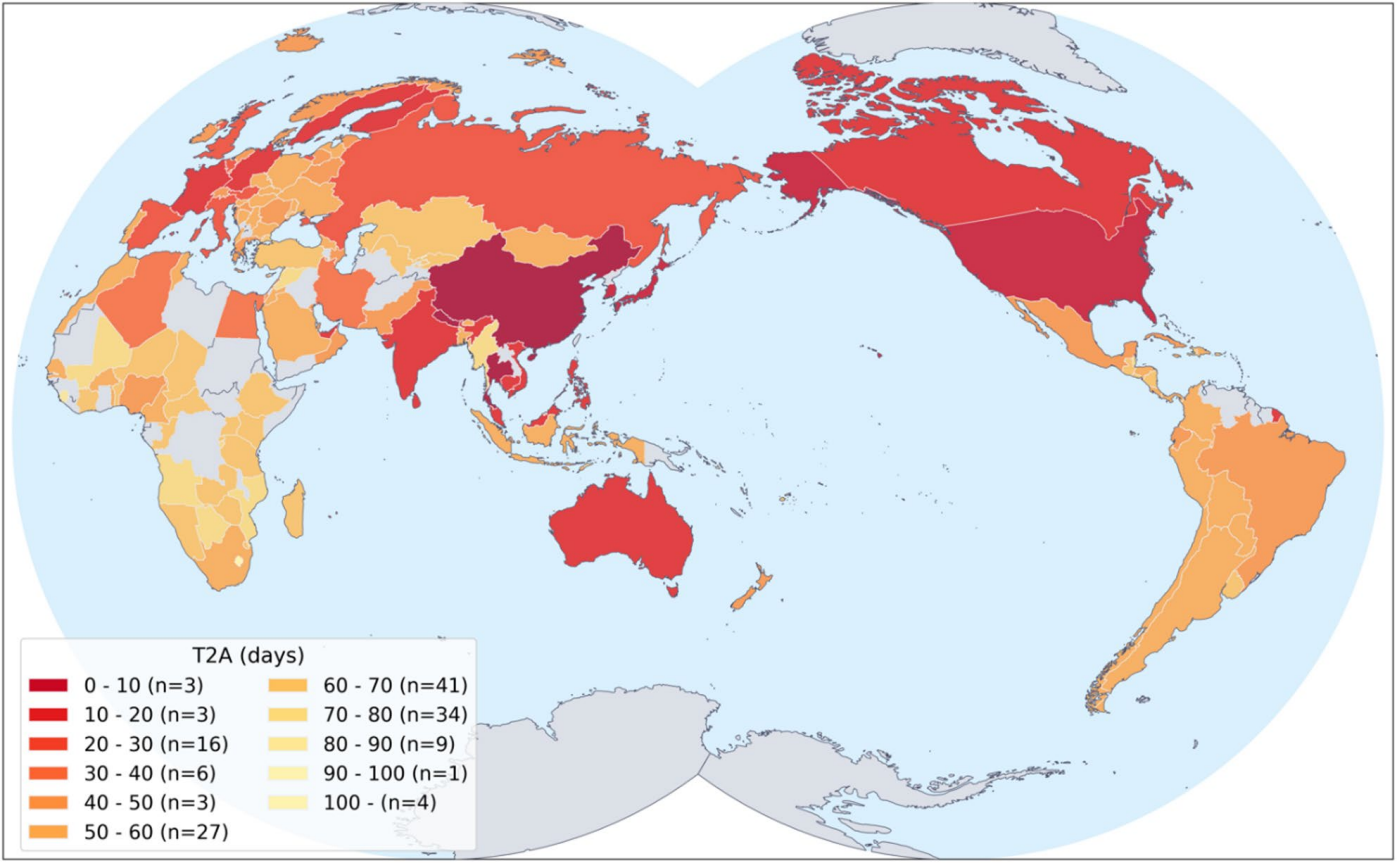
Spatial distribution of the T2A for the first imported COVID-19 case. Countries and regions with darker colors indicate shorter durations until the first imported case, whereas those with lighter colors indicate a longer T2A

Collected from the Johns Hopkins University repository [40], the first imported COVID-19 case dataset provides the date of the first imported COVID-19 case for 229 countries and regions, which was validated using local news reports. The time to arrival (T2A) was calculated as the duration in days between the initial global emergence of COVID-19 on December 31, 2019, and the date the first imported case appeared in each country or region (Fig. 1). The minimum, median, and maximum T2A were 0, 64, and 668 days, respectively.

Country-level socioeconomic status data for 2019 were collected from the World Bank. Five socioeconomic indicators were considered: GDP per capita, life expectancy at birth, imports of goods and services, international tourism, and population. Prepandemic (2019) socioeconomic data were used to reduce the potential impact of pandemic-related events, which could introduce additional uncertainty into the study. S Pardhan and N Drydakis [41] reported that countries with higher GDP per capita tended to have fewer COVID-19 cases. A Notari and G Torrieri [42] found that longer life expectancies and more international tourist arrivals were associated with faster COVID-19 transmission. Moreover, E Bontempi and M Coccia [43] reported that both total imports and exports were strongly correlated with COVID-19 transmission. Nevertheless, H Coşkun, N Yıldırım and S Gündüz [44] reported that population is among the critical factors in disease transmission and that a higher population could lead to faster disease transmission, consistent with the findings of other studies [45, 46].

The international travel restriction data were collected from the Oxford COVID-19 Government Response Tracker (OxCGRT), encompassing 185 countries and regions. This dataset records each country’s daily restrictions for air travelers. Five levels of travel restrictions were classified (ranging from level 0 to level 4): no restrictions, screening arrivals, quarantining arrivals from some or all regions, banning arrivals from some regions, and banning arrivals from all regions or total border closure. For the purpose of the analysis, the international travel restriction of each country and region was selected based on the date when the first case appeared.

Spatially disconnected places within some countries (e.g., French overseas departments) were treated as separate network nodes when air travel records supported distinct flows and when corresponding T2A, socioeconomic, and international travel restriction were available. The reduction from 271 initial air-travel nodes to 147 analytic nodes therefore reflected data intersection across multiple datasets rather than network pruning alone (Table S1 and Table S2). Because missing data were more likely for smaller or less well-documented territories, this selection process may bias the analytic sample toward better-connected countries and regions. Nevertheless, the final subset still captured 92% of the global travel volume in January 2020, supporting a broad characterization of early international mobility structure.

### Formulation of the global country network

The global country network was formulated as a directed and weighted graph $\:G\:=\:(V,\:E,\:W)$, where the nodes ($V$) are countries or regions and the edges ($E$) are the directed international connections with total passenger volumes as the weights ($W$). Fig. S1 provides a visual representation of the graph. The original air travel data contained both direct flights and transfer flights. Direct flights involved a single pair of origin and destination airports, whereas transfer flights involved one or more connecting airports. In this study, the transfer itineraries were decomposed into individual segments, with the same passenger count assigned to each segment between consecutive airports. This simplification assumes no passenger loss at transfer points and equal continuation across itinerary legs; accordingly, it may overstate the intermediary role of major hubs and inflate bridge-related metrics such as betweenness centrality or hub-like transmission proxies. Passenger volumes were then aggregated to the country or region level to focus on global flow patterns. When a region constituted a noncontiguous territory with distinct observed flows, it was retained as an independent node.

### Global country network properties

Two sets of country property measurements were prepared, including node-based network indicators and geographical/socioeconomic indices.

PageRank (PR, Eq. (1)) quantifies the stationary visitation probability of a node in a directed network, that is, the probability that a random walker will be at that node in the long run [47, 48]. In the global country network, we interpret PR as a proxy for passenger arrival probability rather than a direct behavioral measure, because a higher PR indicates that a country or region is more structurally likely to receive flows from other important nodes.

1 $$\:PR\left({C}_{i}\right)=\sum\limits_{{C}_{j}\in\:M\left({C}_{i}\right)}\frac{PR\left({C}_{j}\right)}{L\left({C}_{j}\right)},$$

where $\:M\left({C}_{i}\right)$ is the set of nodes (countries or regions) with inbound connections to node $\:{C}_{i}$ and $\:L\left({C}_{j}\right)$ is the number of outbound connections from node $\:{C}_{j}$.

Betweenness centrality (BC, Eq. (2)) measures the extent to which a node lies on shortest paths between other nodes [49, 50]. In the global country network, BC is interpreted as a proxy for transfer-related connectivity or intermediary prominence, rather than a direct observation of traveler behavior. A higher BC therefore suggests that a country or region occupies a more important bridge-like position within the network structure.

2 $$\:BC\left({C}_{i}\right)=\sum\limits_{k\ne\:i\ne\:j\in\:N}\frac{{\sigma}_{kj}\left(i\right)}{{\sigma}_{kj}},$$

where $\:{\sigma}_{kj}$ is the total number of shortest paths between node $\:{C}_{k}$ and node $\:{C}_{j}$ and $\:{\sigma}_{kj}\left(i\right)$ is the number of shortest paths that pass through a specific node $\:{C}_{i}$. Betweenness centrality is primarily determined by the number of shortest paths via the target node; therefore, we may consider this network property as a measure of transfer-related connectivity. A higher betweenness centrality indicates a greater number of air travelers transferring from the country or region $\:{C}_{i}$ [51–53].

The local clustering coefficient ($CC$, Eq. (3)), or connection density, is a measure of the degree to which nodes in a graph tend to cluster together, indicating that a node has strong connections with other nodes [54]. In the global country network, a higher $\:CC$ represents a higher connection density between the neighboring countries or regions of a targeted country or region [55].

3 $$\:CC\left({C}_{i}\right)=\frac{T\left({C}_{i}\right)}{2\left({\mathrm{deg}}^{{tot}}\left({C}_{i}\right)\right)\left({\mathrm{deg}}^{{tot}}\left({C}_{i}\right)-1\right)-2{\mathrm{deg}}^{\leftrightarrow\:}\left({C}_{i}\right)},$$

where $\:T\left({C}_{i}\right)$ is the number of directed triangles throughout node $\:{C}_{i}$, $\:{\mathrm{deg}}^{{tot}}\left({C}_{i}\right)$ is the sum of the in-degree and out-degree of node $\:\:{C}_{i}$, and $\:{\mathrm{deg}}^{\leftrightarrow}\left({C}_{i}\right)$is the reciprocal degree of node $\:{C}_{i}$.

Because the nodes in this study are countries and regions rather than individual airports, the distance between two nodes was derived from all possible airport pairs between them. Specifically, the distance from node $\:j$ to node $\:i$ was calculated as the average great-circle distance across all airport pairs linking the two nodes. We used this unweighted average distance to represent baseline spatial separation or friction of spread, independent of traffic intensity, because passenger volume was already captured by the network construction and related mobility metrics. Let $\:{A}_{j}$ represent the set of airports in country or region $\:{C}_{j}$, $\:{A}_{i}$ represent the set of airports in country or region $\:{C}_{i}$, and $\:GCdist(x,\:y)$ denote the great circle distance between airports *x* and *y*. The average distance from airport *j* to node *i* can be calculated with Eq. (4):

4 $$\:dist\left({C}_{j},\:{C}_{i}\right)=\frac{1}{\left|{A}_{j}\right|\times\:\left|{A}_{i}\right|}\times\:\sum\limits_{x\in\:{A}_{i}}\sum\limits_{y\in\:{A}_{j}}GCdist\left(x,\:y\right),$$

Capturing the geographical distance between a pair of countries or regions could enrich the understanding of the geographical proximity effect, which may improve the effectiveness of disease transmission prevention policies. The average distance to a specific country or region $\:I\:$ can then be calculated as the average distance from all the other nodes to node *i*:

5 $$\:av{eDist}_{\left({C}_{i}\right)}=\frac{1}{N-1}\sum\limits_{j\ne\:i}dist\left({C}_{j},\:{C}_{i}\right).$$

### Spatial spreader, receiver, and transmission potential

Hubs and authorities are key components of the Hyperlink-Induced Topic Search (HITS) algorithm, which recursively evaluates how nodes are positioned within a directed network [56, 57]. In this study, the hub score represents a country’s or region’s structural tendency to connect to important receiver nodes, whereas the authority score represents its tendency to receive links from important spreader nodes. Unlike simple inflow or outflow totals, these recursive measures capture a node’s relational role within the broader topology of the international flight network. We therefore interpret the hub score as a proxy for spatial spreader potential and the authority score as a proxy for spatial receiver potential. The sum hub and authority scores obtained from the HITS algorithm were utilized to evaluate each node’s spatial transmission potential (STP), allowing for a comprehensive assessment of their ability to influence disease spread within the global country network.

### Associations between global country network properties and disease transmission

We explored the relationships between global country network properties (passenger arrival probability, transfer-related connectivity, connection density, spatial spreader potential, and spatial receiver potential) and disease spread (T2A) using a Poisson generalized linear model (GLM). Because T2A represents the number of days until the first imported COVID-19 case, a Poisson GLM was selected. The socioeconomic variables were included to assess whether macroscale development indicators explained additional variation beyond mobility-related metrics. Prior to model fitting, continuous predictors were standardized to z-scores to improve coefficient comparability and numerical stability. In this study, T2A was the dependent variable, whereas the network properties and socioeconomic indicators were treated as independent variables. The model can be expressed as follows:

6 $$\begin{aligned}\:{\mathrm{l}\mathrm{n}(y}_{T2A}^{{C}_{i}})&\:={\beta\:}_{GDP}{x}_{GDP}^{{C}_{i}}+{\beta\:}_{LEB}{x}_{LEB}^{{C}_{i}}+{\beta\:}_{IM}{x}_{IM}^{{C}_{i}}\\ &\quad+{\beta\:}_{TOUR}{x}_{TOUR}^{{C}_{i}}+{\beta\:}_{POP}{x}_{POP}^{{C}_{i}}+{\beta\:}_{ITR}{x}_{ITR}^{{C}_{i}}{+\beta\:}_{Dist}{x}_{Dist}^{{C}_{i}}\\ &\quad+{\beta\:}_{PAP}{x}_{PAP}^{{C}_{i}}+{\beta\:}_{TSC}{x}_{TSC}^{{C}_{i}}+{\beta\:}_{CD}{x}_{CD}^{{C}_{i}}+\epsilon\:\end{aligned}$$

where $\:{x}_{GDP}^{{C}_{i}}$, $\:{x}_{LEB}^{{C}_{i}}$,$\:\:{x}_{IM}^{{C}_{i}}$,$\:\:{x}_{TOUR}^{{C}_{i}}$, and$\:{\:x}_{POP}^{{C}_{i}}$ are GDP per capita, life expectancy at birth, the importation of goods and services, international tourism, and the population of each node $\:{C}_{i}$, respectively. $\:{x}_{ITR}^{{C}_{i}}$ and $\:{x}_{Dist}^{{C}_{i}}$ represent international travel restrictions and inverse weighted geographical distances between nodes. Notably, travel restrictions and socioeconomic status were modeled as independent covariates in the GLM to assess their additional explanatory value alongside network features. For global country network properties, ${x}_{PAP}^{{C}_{i}}$, ${x}_{TSC}^{{C}_{i}}$, and $\:{x}_{CD}^{{C}_{i}}$ represent the passenger arrival probability (measured by PageRank), transfer-related connectivity (betweenness centrality), and connection density (clustering coefficient), respectively. In the evaluation of the model, we used the ratio of the deviance to the number of observations to test for overdispersion in a Poisson GLM. A ratio greater than 1 indicates the presence of overdispersion. We corrected for overdispersion using a negative binomial GLM initially, but if overdispersion still existed, a Poisson GLM with the sandwich estimator method was adopted to obtain robust coefficient estimates.

### Delineation of spatial epidemic prevention zones

Spatial epidemic prevention zones (SEPZs) represent the zones of nodes with strong internal connections and weak external connections. This definition means that internal flows within SEPZs are dense, whereas external flows within other zones are sparse. Therefore, closing SEPZ borders during an epidemic would have a weaker effect on people’s daily lives within the same SEPZ. Due to the strong internal flow within SEPZs, rapid virus spread is anticipated during an epidemic. Thus, an effective strategy would be to focus on controlling the borders of SEPZs and implementing measures to prevent the importation of the disease into these SEPZs. This approach could help contain the spread of the disease while minimizing disruptions to daily life within the SEPZs.

In this study, Infomap [58] was used to delineate the SEPZs, which are the network communities of countries and regions, based on the global country network with total passenger volume flows between nodes. Infomap is a community detection method suitable for directed and weighted networks [59, 60]. Infomap processes weighted graphs by incorporating edge weights into its random walk process. In a weighted graph, the probability of moving from one node to another is proportional to the edge weight. Edges with higher weights are traversed more frequently, hence influencing the random walk process. Additionally, since the random walker spends more time on high-weight edges, the nodes connected by these edges are more likely to be grouped into the same community. This approach allows Infomap to effectively identify tightly connected communities within a network by minimizing the description length of the random walker’s movements, resulting in optimal community partitioning [58]. Through many iterations, Infomap determines the optimal grouping results by minimizing a loss function defined as follows:

7 $$\:L\left(M\right)={q}_{\curvearrowright\:}H\left(Q\right)+\sum\limits_{i=1}^{m}{p}_{\circlearrowright}^{i}H\left({P}^{i}\right),$$

where $\:{q}_{\curvearrowright\:}$ is the probability of exiting a community $i$, and $\:{p}_{\circlearrowright\:}^{i}$ is the probability of visiting the nodes within a community $\:i$. $\:H\left(Q\right)$ is the frequency-weighted average entropy of cross-communities, and $\:H\left({P}^{i}\right)$ is the frequency-weighted average entropy within a community $\:i$. This loss function enables Infomap to identify the most relevant communities within the global country network based on passenger volume flows, ultimately facilitating the delineation of SEPZs for further analysis.

### Disease spread risk stratification and scenarios for determining the disease control policy

Both the SEPZs and the STP values were considered to assess the risk of disease spread across countries and regions. Nodes were categorized into four distinct levels based on STP quartiles derived from the composite STP score, which was calculated from the combined hub and authority scores: (1) top level (STP-Q1, 0–25%); (2) second level (STP-Q2, 25–50%); (3) third level (STP-Q3, 50–75%); and (4) fourth level (STP-Q4, 75–100%). These categories were established to guide the development of targeted disease control scenarios. Given the STP levels, two scenarios were discussed when an outbreak occurred in a country or region (outbreak node):

Scenario S1: What level of measures should be implemented for a target country or region located within the same SEPZ as the outbreak node?

Scenario S2: What level of measures should be implemented for a target country or region located in a different SEPZ?

By integrating SEPZs and STP classifications, this study aimed to provide a framework for stratifying the disease spread risk and informing policy decisions to mitigate the spread of diseases across countries and regions. This approach enables policymakers to develop tailored strategies to address various risk levels and scenarios effectively.

### Identification of temporal infection clusters

Temporal infection clusters (TICs) were identified using k-means clustering to analyze T2A similarity between nodes (countries and regions). In this study, T2A served as a primary feature for investigating TICs during the first wave of the COVID-19 pandemic at each node. K-means clustering was applied using the following mathematical definition:

8 $$\:\underset{\boldsymbol{S}}{\text{arg min}}\sum\limits_{i=1}^{k}\sum\limits_{T\in\:{S}_{i}}{\lVertT-{u}_{i}\lVert}^{2},\:where\:{u}_{i}=\frac{1}{\left|{S}_{i}\right|}\sum\limits_{T\in\:{S}_{i}}T$$

where $\:T=({t}_{1},{t}_{2},\dots\:,\:{t}_{n})$ is the set of T2A data in each temporal infection cluster and $\:S=\left\{{S}_{1},{S}_{2},\dots\:,\:{S}_{k}\right\}$ is the set of temporal infection clusters. The objective function is to minimize the differences in T2A between nodes in a TIC. We further adopted the elbow method to determine the most suitable number of TICs:

9 $$\:\underset{\boldsymbol{S}}{\text{arg min}}\sum\limits_{i=1}^{k}\left|{S}_{i}\right|Var\left({S}_{i}\right),$$

where $\:\left|{S}_{i}\right|$ is the size of $\:{S}_{i}$. The best number of TICs was determined by the turning point of the trend plot between the number of TICs and the within-cluster sum of squares. By utilizing k-means clustering, this study aimed to reveal the initial patterns of COVID-19 spread across different nodes and identify clusters with distinct temporal infection characteristics.

## Results

### Relationships between the time to arrival and global country network properties

The relationships between global country network properties and disease transmission were assessed using a Poisson GLM with sandwich covariance correction (Table 1). Passenger arrival probability (PageRank) (*β=*–0.210, 95% CI=–0.417 to − 0.002, *p* = 0.047) and connection density (clustering coefficient) (*β=*–0.228, 95% CI=–0.356 to − 0.101, *p* = < 0.001) were significantly associated with a shorter time to arrival (T2A) of the first imported case, indicating that greater structural exposure and denser local connectivity accelerated early importation. Transfer-related connectivity (betweenness centrality) (*β=*–0.097, 95% CI=–0.198 to − 0.003, *p* = 0.058) showed only limited evidence of an association with T2A and should therefore be interpreted cautiously. In contrast, greater average geographical distance (*β=*–0.231, 95% CI=–0.424 to − 0.039, *p* = 0.018) between countries was associated with a longer T2A, suggesting that spatial separation still exerted a friction effect on early spread despite the global reach of the airline network.

**Table 1:** The Poisson GLM results with sandwich correction

	Coefficient		Std. err.	*p* value	[0.025	0.975]
Constant	4.093	0.035		< 0.001	4.024	4.162
GDP per capita	–0.035	0.055		0.521	–0.143	0.072
Life expectancy	0.057	0.048		0.228	–0.036	0.151
Importation	–0.072	0.155		0.644	–0.375	0.232
International tourist arrivals	0.047	0.052		0.373	–0.056	0.149
Population	–0.189	0.200		0.344	–0.581	0.203
International travel restriction	0.190	0.132		0.152	–0.070	0.449
Passenger arrival probability	–0.210	0.106		0.047	–0.417	–0.002
Transfer-related connectivity	–0.097	0.051		0.058	–0.198	0.003
Connection density	–0.228	0.065		< 0.001	–0.356	–0.101
Average geographical distance	–0.231	0.098		0.018	–0.424	–0.039

### Identification of temporal infection clusters

The k-means clustering method initially identified five temporal infection clusters (TICs). Three countries, the Kingdom of Tonga, the Republic of Vanuatu, and the Solomon Islands, exhibited extremely long T2A durations (more than 200 days) and formed two sparse cluster in the five TICs. For interpretability, these three countries were subsequently merged into a single TIC, yielding four TICs in the final grouping result (Fig. 3). The TIC analysis was included to summarize heterogeneity in early importation timing and to assess whether temporal groupings corresponded to the network-derived SEPZs and STP hierarchy. The first TIC comprised nodes in East Asia, South Asia, West Europe, Australia, and North America (*n* = 28), with a T2A of less than 40 days. The second TIC included approximately half of the nodes (*n* = 67), with a T2A range of 40–70 days. The third TIC (*n* = 49) had a T2A range of 70–140 days, whereas the fourth TIC consisted of the three countries with a T2A exceeding 200 days. These TICs provided valuable insights into the temporal patterns of disease spread across different regions and countries during the first wave of the COVID-19 pandemic.

**Fig. 2:**
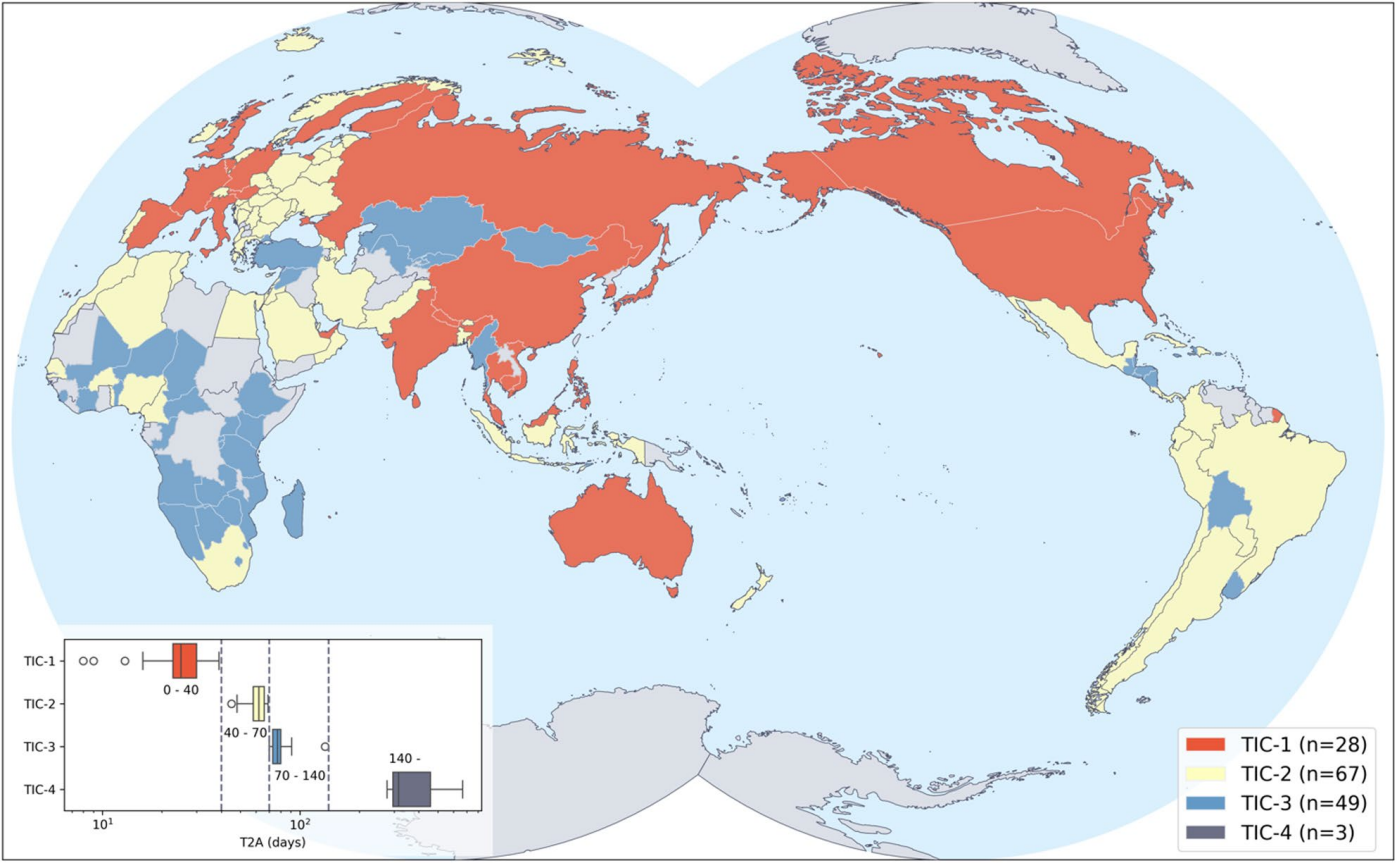
Spatial distribution of the four TICs identified during the COVID-19 pandemic. The inset displays a box plot of the T2A for each TIC, illustrating the range and variation in the T2A across clusters

### Delineation of spatial epidemic prevention zones

Using the Infomap algorithm, five SEPZs across 147 nodes were delineated (Fig. 2). SEPZ-3 emerged as the largest SEPZ, encompassing 56 nodes in Northwest Africa, Europe, and Central Asia. SEPZ-4 contained 27 nodes located in North America and South America. SEPZ-5 contained 24 nodes in Central to South Africa. The Asia–Pacific region formed SEPZ-1 with 23 nodes, and SEPZ-2, the smallest SEPZ, comprised 17 West and South Asian nodes. These SEPZs provide a comprehensive spatial representation of node groupings, reflecting the connectivity of countries and regions and the potential for disease transmission within the global country network.

**Fig. 3:**
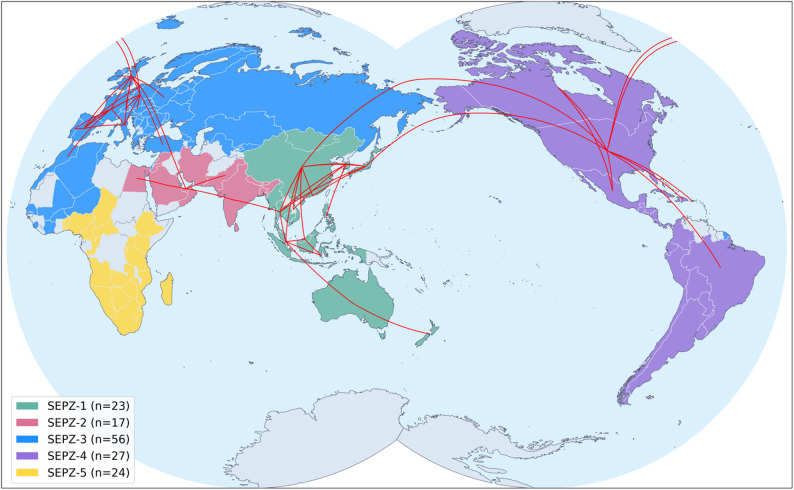
Spatial distribution of the five SEPZs. The lines represent great circle lines whose passenger volume is two standard deviations greater than the mean volume, highlighting significant connections between countries and regions within the global country network

### Spatial dynamics of disease transmission

By linking SEPZ groupings and STP quartiles, this framework identifies countries and regional pathways associated with higher early importation risk and can support more adaptive border-control scenario planning. The HITS algorithm was applied to the global country network to analyze the spatial spreader potential within a TIC. The results indicated a positive correlation between spatial receiver potential and spatial spreader potential, suggesting that countries with stronger structural exposure as receivers also tended to occupy more prominent spreading positions (Fig. S2).

**Fig. 4:**
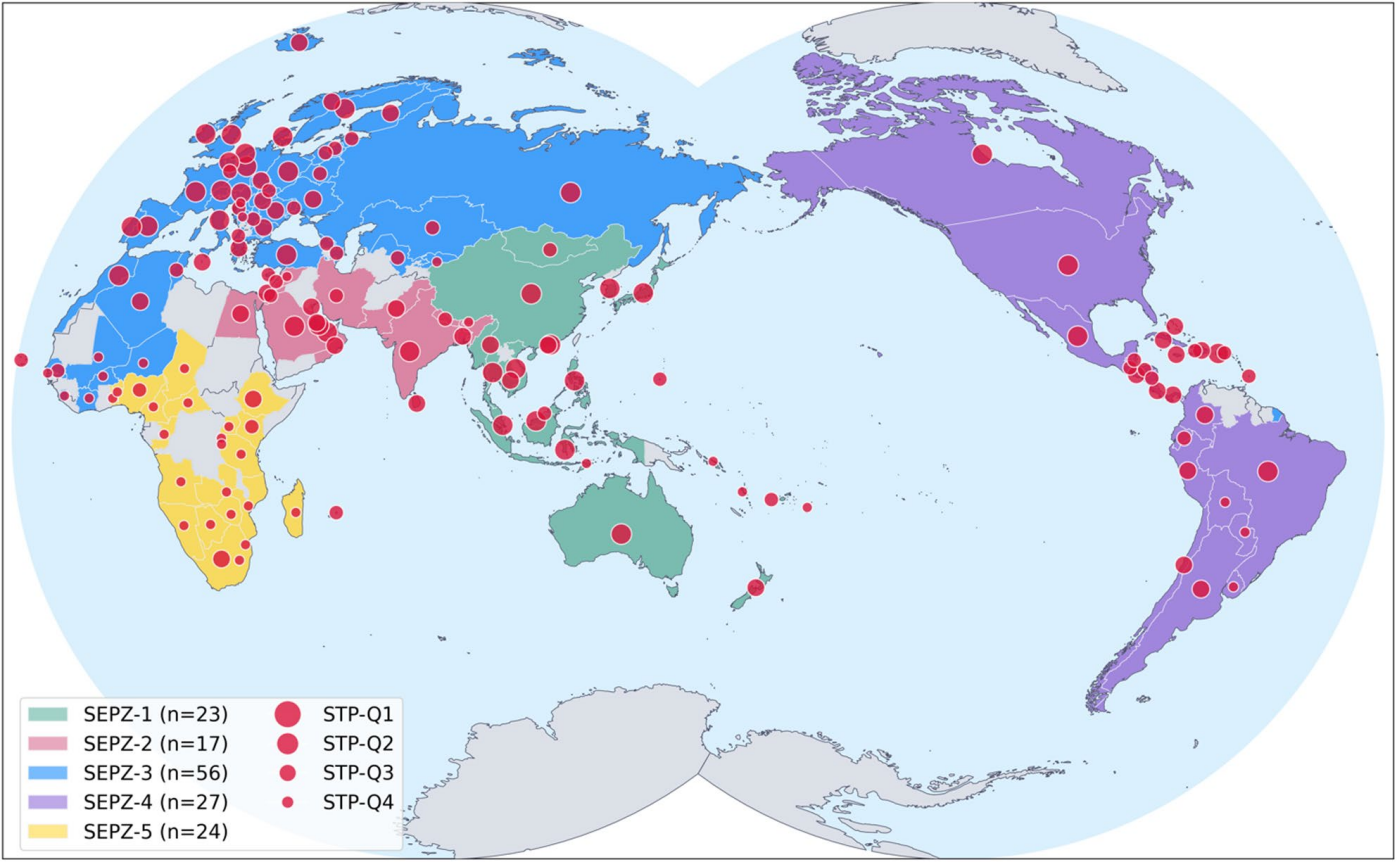
STP distribution across countries and regions within each SEPZ. The STP is categorized into four quartiles (STP-Q1, STP-Q2, STP-Q3, and STP-Q4) and mapped with the SEPZs, illustrating the spatial dynamics of disease transmission

Figure 4 presents the STP categorized into four quartiles (STP-Q1, STP-Q2, STP-Q3 and STP-Q4) mapped alongside the SEPZ distribution. The spatial distribution demonstrates that nodes with higher STPs are usually surrounded by nodes with relatively high STPs. Nodes identified in the STP-Q1 group generally exhibit high-density connections within their respective SEPZs, resulting in accelerated disease transmission because of the significant number of passenger arrivals. Furthermore, STP-Q1 nodes also exhibit strongly connections to other STP-Q1 nodes compared with the STP-Q2, STP-Q3, and STP-Q4 groups. Nodes in the STP-Q2 and STP-Q3 groups have more connections with regional nodes, providing a disease transmission pathway linked from STP-Q1 to nodes in other quartiles. This pattern facilitates disease transmission propagation from larger nodes to smaller nodes. In contrast, the STP-Q4 group has limited connections with regional nodes, making it more difficult for the disease to spread across nodes within this group.

**Fig. 5:**
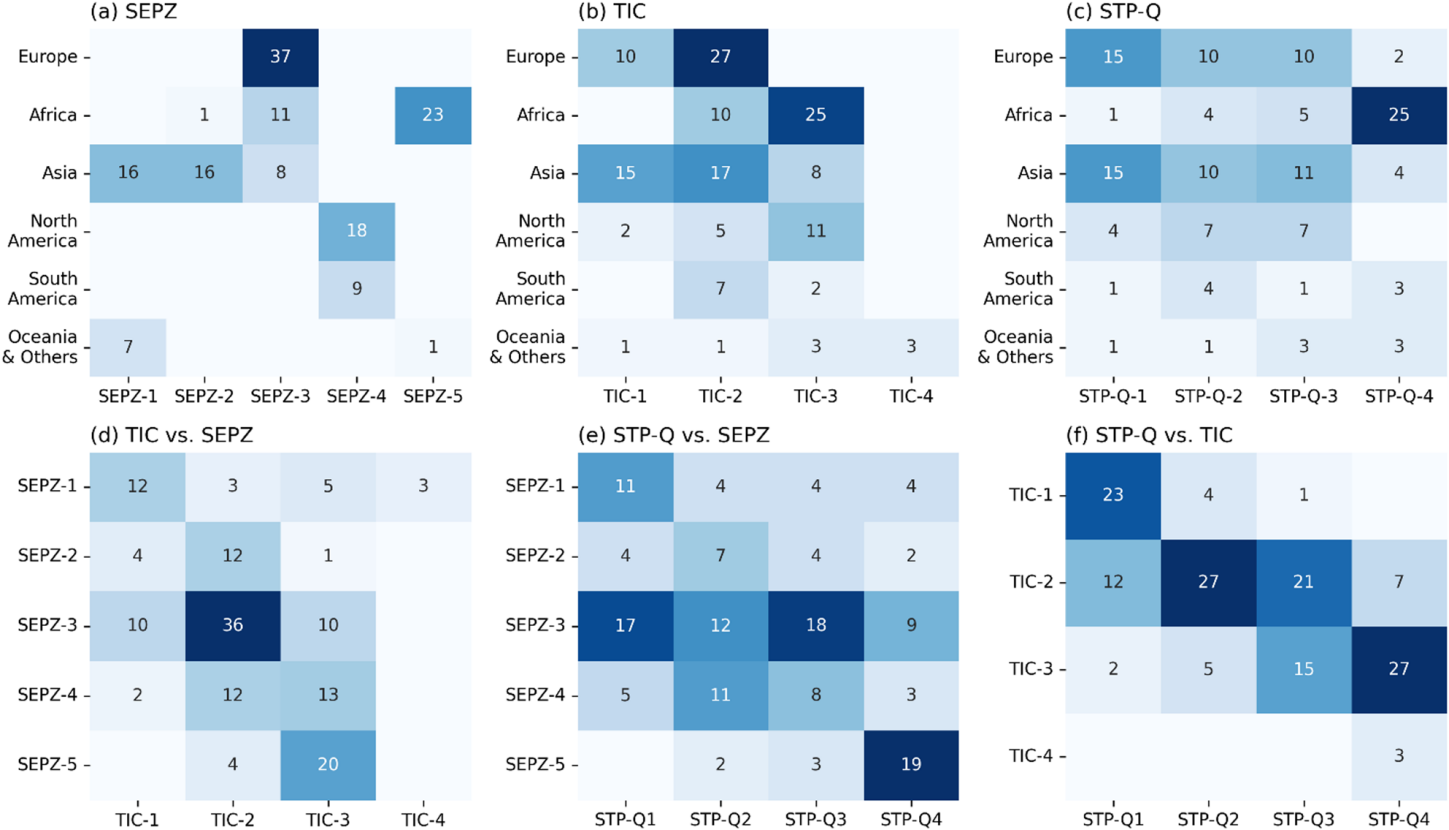
Number of nodes within each pair of the four grouping dimensions: continents, spatial epidemic prevention zones (SEPZs), temporal infection clusters (TICs), and spatial transmission potential quartiles (STP-Qs). Top row: Number of nodes on each continent quantified by (**a**) SEPZ, (**b**) TIC, and (**c**) STP-Q. Bottom row: Number of nodes in each (**d**) SEPZ quantified by TIC, (**e**) SEPZ by STP-Q, and (**f**) TIC by STP-Q. Blank cells indicate zero nodes

In Fig. 5, the number of nodes falling in each pair of the four grouping dimensions was counted. The top row ((a) to (c)) shows the distributions of the five SEPZs, four TICs, and four STP quartiles across the six continents/Oceania, as previously described in Figs. 2, 3 and 4. The bottom row ((d) to (f)) presents the counts of nodes in the combinations of SEPZ, TIC, and STQ quartiles. For further context, Supplementary Table S3 lists the top 20 nodes with the highest STP, while Table S4 lists the nodes with the four STP levels within each SEPZ. These tables provide additional insights into spatial transmission dynamics across different countries and regions.

Figure 5(d) depicts the number of nodes in each SEPZ quantified by TIC, highlighting that most SEPZs experienced a T2A within the same TIC, except for SEPZ-4, which had a T2A crossing between TIC-2 and TIC-3. SEPZ-1 was impacted by the pandemic during the first 40 days (TIC-1). Most nodes in SEPZ-2 and SEPZ-5 were affected by TIC-2 (40 to 70 days) and TIC-3 (70 to 140 days), respectively. The majority of SEPZ-3 (36 out of 56 nodes) were infected during TIC-2; 10 of the nodes in SEPZ-3 occurred in TIC-1, and another 10 nodes occurred in TIC-3. This pattern suggests that strong internal flow could allow the virus to spread quickly within a SEPZ. The exception of SEPZ-4 implies a possible hierarchical structure within North and South American countries.

An examination of the number of nodes in each SEPZ quantified by the STP quartile (Fig. 5(e)) revealed similar observations: most nodes in SEPZ-1, SEPZ-2, and SEPZ-5 fell into STP-Q1, STP-Q2 and STP-Q4, respectively. This finding indicated a hierarchical structure within the SEPZ with respect to the STP. SEPZ-3, which was composed mainly of nodes in Europe, North Africa, and Central Asia, had 56 nodes distributed across STP-Q1 to STP-Q3, suggesting an internal network hierarchical structure; i.e., 30% of the nodes had a higher STP quartile, and the other 30% had a lower STP quartile. Similarly, 40% of the nodes of SEPZ-4 (*n* = 11) were in STP-Q2, and 30% (*n* = 8) were in STP-Q3, emphasizing the complex internal network structure that can occur within SEPZs, potentially posing challenges for the establishment of disease control policies.

A clear hierarchical relationship was observed in the number of nodes in each TIC quantified by the STP quartile (Fig. 5(f)). Nodes with a short T2A (TIC-1, within the first 40 days) tended to have a higher STP and were concentrated in STP-Q1. TIC-2 nodes were concentrated mainly in STP-Q2 and STP-Q3, whereas TIC-3 nodes were found primarily in STP-Q3 and STP-Q4. The three TIC-4 nodes exhibited a low STP. This pattern suggests that nodes with a higher STP were associated with a shorter T2A and therefore with higher early importation risk than nodes with a lower STP. Thus, the STP quartile may be a useful tool for scenario-based disease control planning.

### Establishment of hierarchical disease control policies

Minimizing the importation risk while preserving essential international mobility, maintain stable international trade, and sustain tourist arrivals is a central challenge in cross-border disease control. In this study, we propose a four-level risk stratification framework for border-control decision support (Table 2), informed by policy patterns observed during the COVID-19 pandemic [61–63]. For a target node, the appropriate level of control may vary across incoming flows according to the epidemic status of both the target node and the origin mode. We therefore present the resulting policy levels as structured scenarios to support planning and comparison, rather than as universally prescriptive rules.

**Table 2:**
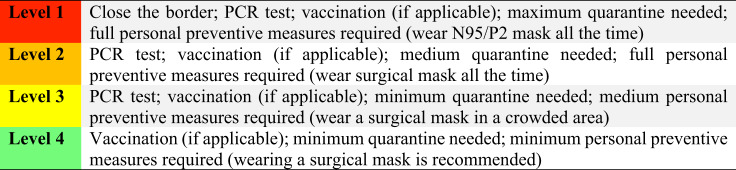
The four levels of disease control policies for various origin nodes

Using the information from the SEPZ and the quartiles of the STP, a hierarchical disease control policy was developed for the two epidemic scenarios. Given a target node (country or region), two scenarios could be considered: (1) the current outbreak occurs in a node within the same SEPZ (S1 series), and (2) the outbreak occurs in the nodes in another SEPZ (S2 series). Under scenario S1, when the outbreak occurs within the same SEPZ, the disease control policies that the target node could apply are listed in Table 3. Four subscenarios (S1-1 to S1-4) indicate the policies that the target node can apply when the outbreak occurs in nodes with different quartiles of STP levels. For example, if an outbreak occurs in an STP-Q1 node within the same SEPZ (scenario S1-1), the target node should apply a level 1 policy to all other origin nodes in the same SEPZ, as well as the STP-Q1 and STP-Q2 nodes in other SEPZs. This approach is needed because many air travelers from the outbreak node (STP-Q1) can travel to any other node with a higher flight frequency, especially to the STP-Q1 and STP-Q2 nodes. The discrepancy in disease control policy levels between the S1-2 and S1-3 scenarios reflects the fact that the STP-Q2 and STP-Q3 nodes mainly connect to the STP-Q1 nodes.

**Table 3:**
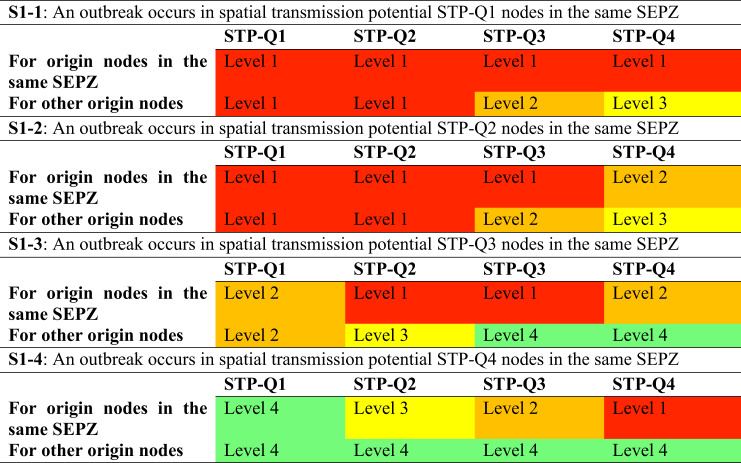
The hierarchical disease control policy for the first scenario is when the outbreak occurs in the same SEPZ as the target node

Under scenario S2, when the outbreak occurs in another SEPZ, the suggested policy levels are listed in Table 4. Scenario S2 is divided into four subscenarios (S2-1 to S2-4) according to the STP quartiles (STP-Q1 to STP-Q4) of the outbreak node. Furthermore, the origin nodes in other SEPZs are classified into two conditions: those in SEPZs with an outbreak node and those without an outbreak node. For instance, if the target node is located in an SEPZ without an outbreak (scenario S2) and the outbreak occurs in an STP-Q1 node (scenario S2-1), the target node can apply a level 1 policy for the STP-Q1 and STP-Q2 origin nodes in the same SEPZ; level 3 and 4 policies for the STP-Q3 and STP-Q4 origin nodes in the same SEPZ, respectively; the strictest policy (level 1) for those origin nodes in the SEPZ with an outbreak; and slightly looser policies for other countries and regions in the nonoutbreak SEPZ.

**Table 4:**
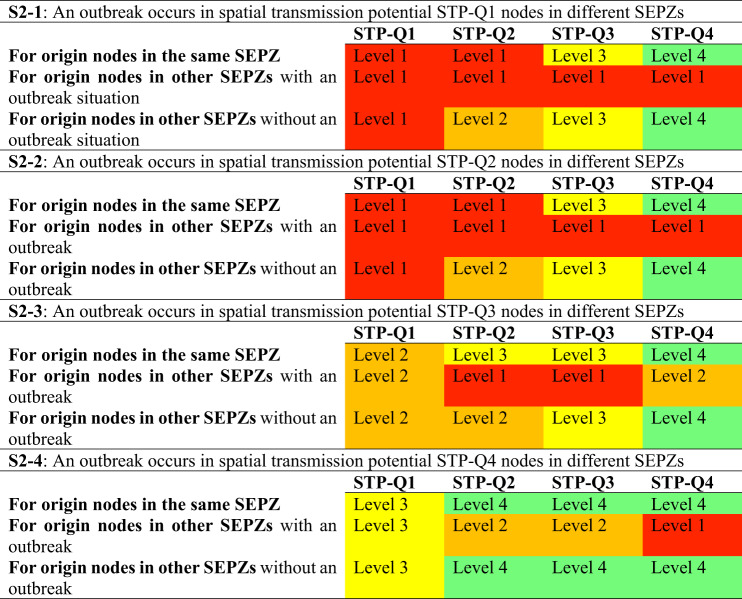
The hierarchical disease control policy for the second scenario

## Discussion

In this study, we examined the relationships between global country properties and early-stage COVID-19 transmission and identified SEPZs and TICs to assess country-specific early importation risk. Our findings revealed that the passenger arrival probability and connection density were associated with earlier arrival of the first imported case, whereas longer inter-country distance was associated with delayed arrival. These results indicate that mobility-based network metrics capture important features of cross-border spread and that spatial separation still matters even in a highly connected airline system. We further delineated five SEPZs to represent major connectivity structures between countries and regions and calculated STP values to characterize variation in transmission-related prominence within and across zones.

Complex network analysis has proven effective for studying how the COVID-19 pandemic interacted with air transportation and regional mobility systems [37, 55, 64, 65]. Whereas some previous studies emphasized transfer-related connectivity (betweenness centrality) as a key determinant of spread [53]. Our results suggest that passenger arrival probability and connection density were more informative for explaining the timing of first imported cases in this international dataset, which is consistent with previous findings [55, 66–68].

As local outbreaks strain medical resources and economic development, countries implement varying levels of international travel restrictions to mitigate imported cases and reduce local transmission [2, 11]. Although prior studies reported that such restrictions can provide short-term benefits [69], our findings suggest that board travel controls alone may not fully prevent early importation when countries remain highly connected through regional mobility systems. Geographical proximity retained a measurable association with T2A, indicating that neighboring countries with frequency interactions can remain vulnerable even when long-haul transmission routes are also present [70]. In this context, macroscale socioeconomic indicators showed limited explanatory power for early importation timing compared with mobility-based metrics in our model.

Early-stage disease transmission makes it difficult to calibrate appropriate prevention measures and screening intensity in a timely and proportionate manner. Delineating global SEPZs could help organize countries and regions into functionally connected groups that are more relevant than equal-radius or purely adjacency-based zoning. While S Hsiang, D Allen, S Annan-Phan, K Bell, I Bolliger, T Chong, H Druckenmiller, LY Huang, A Hultgren and E Krasovich [69] noted the initial benefits of travel restrictions, their effectiveness diminishes once local outbreaks occur. Studies have shown that connectivity-based zoning can be more effective than equal-radius buffer zoning, as it captures disease transmission patterns and optimizes control measures by covering potential risk locations [19, 21]. The World Health Organization recommends considering the geographical distribution and movement of confirmed cases for epidemic zoning [71]. By disrupting high-risk routes, the spread of imported cases and local outbreaks could be reduced [72]. In this study, we delineated global SEPZs based on the flight frequency and geographical proximity to identify countries and regions with strong interconnectivity, i.e., the SEPZs. Although several high-frequency long-haul cross-continent flights occurred, the delineated SEPZs were mostly from geographically adjacent countries and regions. Numerous airports and high passenger arrivals in large countries often have strong regional connections, resulting in more short-haul flights than long-haul flights [73]. Therefore, air transportation in SEPZs could effectively outline the combination of highly interconnected countries or regions, hence expediting the policymaking process for early-stage national or international pandemic prevention and control.

A detailed assessment of the disease transmission risk is crucial to support policymaking. Previous studies have established social network indicators to characterize the risk of disease spread [38, 53, 55]. However, most social network indicators consider only mono-directional connections and small-range surrounding connections, limiting their representative and explanatory power. Compared with simple traffic totals or mono-directional indicators, HITS-based hub and authority scores capture how countries are embedded within the broader directed topology of the airline network. In other words, they distinguish structural spreader-like and receiver-like roles rather than merely counting passengers. In our framework, combining these two dimensions into STP provided a concise way to stratify countries according to their relative transmission-related prominence while retaining the relational logic of the global network (Table S3 and Fig. S1).

Based on SEPZ and STP level, we developed a four-level policy stratification framework intended for scenario planning rather than universal prescription [7, 62, 74, 75]. Both S1-series and S2-series scenarios reflect how risk may differ according to whether the outbreak node is located within the same SEPZ as the target node or in a different SEPZ, as well as according to the target node’s STP level. Because pathogens vary in transmissibility, severity, and incubation characteristics, and because countries differ in capacity and governance context, these policy scenarios should be interpreted as adaptable decision-support heuristics rather than fixed recommendations. Their value lies in structuring evidence-based discussion among governments and public health organizations about proportional early-stage responses under networked global mobility. While achieving consensus and coordinating policy actions across nations is challenging, our findings emphasize the interconnected nature of global air travel and the need for collective strategies. Prolonging the epidemic peak and reducing the number of confirmed cases provides more time for medical resource preparation and vaccine development [76]. Effective epidemic control allows for sustained national productivity, functionality, and operation, reducing the social and psychological impacts of a pandemic. Greater control results in fewer travel restrictions, lower mortality rates, lower unemployment rates [77], and a decrease in mental health issues such as depression and anxiety.

This study has four main limitations. First, although 147 countries and regions were included, missing nodes may have influenced the delineation of epidemic prevention zones and the estimated network properties; because missing data were more likely among smaller or less connected areas, some bias toward highly connected countries may remain. Second, the analysis relied on January 2020 flight data as a static representation of the air network and therefore did not capture subsequent temporal restructuring of routes and passenger volumes during the pandemic. Third, because empirical data on infected passengers were unavailable, we assumed that infection risk was proportional to total passenger volume, and the decomposition of transfer itineraries may have overstated the structural role of major transit hubs. Fourth, the effectiveness of prevention zones could not be fully validated because the exact origins of imported cases remain uncertain. Finally, by focusing on international flows, our analysis did not examine subnational heterogeneity within large countries.

## Conclusions

This study demonstrates that global flight networks strongly influenced the early cross-border spread of COVID-19, with passenger arrival probability and connection density emerging as key correlates of first-case importation timing. Countries and regions located in more prominent SEPZ and STP groupings were generally associated with higher early importation risk. Building on these patterns, we propose a four-level hierarchical control framework as a spatially informed decision-support tool for early-stage cross-border risk assessment and scenario planning. Rather than prescribing a single universal policy, the framework provides shared network-based references that may help governments compare risk, prioritize surveillance, and discuss proportional interventions under future outbreak conditions. Future research should extend the analysis to dynamic flight networks, additional pathogens, traffic-weighted distance, other transport modes, and simulation-based evaluation of alternative control scenarios.

## Supplementary Information

Below is the link to the electronic supplementary material.

Supplementary Material 1.

## Data Availability

The data and code that support the findings of this study are available at https://figshare.com/s/b0151c2267bcac8b6ea4. Please read the Instruction.pdf and follow the instructions to reproduce the results.
