# Supplementary material for: Understanding the role of international flight networks in disease spread: spatial epidemic prevention zones and hierarchical disease control policies
Source: Int J Health Geogr. 2026 May 18;25:39. doi: 10.1186/s12942-026-00471-9 (PMC13397806; doi:10.1186/s12942-026-00471-9)
Supplement: Supplementary file 1 — Supplementary Material 1. [file 12942_2026_471_MOESM1_ESM.docx]

| **Table S1** The exclusion criteria and corresponding countries or regions | |
| --- | --- |
| **Exclusion Criteria** | **List of Countries or Regions** |
| After eliminating US Domestic Provinces or Regions (228) | AFG, ALB, DZA, ASM, AGO, AIA, ATG, ARG, ARM, ABW, AUS, AUT, AZE, BHS, BHR, BGD, BRB, BLR, BEL, BLZ, BEN, BMU, BTN, BOL, BES, BIH, BWA, BRA, BRN, BGR, BFA, BDI, KHM, CMR, CAN, CPV, CYM, CAF, TCD, CHL, CHN, TWN, CXR, CCK, COL, COM, COG, COD, COK, CRI, CIV, HRV, CUB, CUW, CYP, CZE, DNK, DJI, DMA, DOM, ECU, EGY, SLV, GNQ, ERI, EST, SWZ, ETH, FLK, FRO, FJI, FIN, FRA, GUF, PYF, GAB, GMB, GEO, DEU, GHA, GIB, GRC, GRL, GRD, GLP, GUM, GTM, GIN, GNB, GUY, HTI, HND, HKG, HUN, ISL, IND, IDN, IRN, IRQ, IRL, ISR, ITA, JAM, JPN, JOR, KAZ, KEN, KIR, PRK, KOR, KWT, KGZ, LAO, LVA, LBN, LSO, LBR, LBY, LTU, LUX, MAC, MKD, MDG, MWI, MYS, MDV, MLI, MLT, MHL, MTQ, MRT, MUS, MYT, MEX, FSM, MDA, MCO, MNG, MNE, MSR, MAR, MOZ, MMR, NAM, NRU, NPL, NLD, NCL, NZL, NIC, NER, NGA, NIU, NFK, MNP, NOR, OMN, PAK, PLW, PAN, PNG, PRY, PER, PHL, POL, PRT, PRI, QAT, REU, ROU, RUS, RWA, BLM, SHN, KNA, LCA, MAF, SPM, WSM, STP, SAU, SEN, SRB, SYC, SLE, SGP, SVK, SVN, SLB, SOM, ZAF, SSD, ESP, LKA, VCT, SDN, SUR, SWE, CHE, SYR, TJK, TZA, THA, TLS, TGO, TON, TTO, TUN, TUR, TKM, TCA, TUV, USA, UGA, UKR, ARE, GBR, URY, UZB, VUT, VEN, VNM, VGB, VIR, WLF, YEM, ZMB, ZWE |
| No socioeconomics  (74) | AFG, AND, AIA, ATG, ABW, BMU, BES, VGB, CYM, CUW, COD, DJI, DMA, GNQ, ERI, FRO, FLK, GUF, GAB, GHA, GIB, GRL, GRD, GLP, GGY, GIN, GUY, IRQ, IMN, JEY, RKS, LAO, LBR, LBY, LIE, MWI, MTQ, MRT, MYT, MCO, MSR, NCL, MNP, PNG, REU, KNA, BLM, LCA, VCT, SMR, STP, SYC, SXM, SOM, SSD, SDN, SUR, TWN, TTO, TCA, VAT, VEN, SHN, TJK, ALA, SPM, COK, NIU, NFK, PRK, PLW, TUV, WLF, YEM |
| No Social Network Indicators  (82) | AFG, AND, AIA, ATG, ARM, ABW, BMU, BES, VGB, CYM, COM, CUW, COD, DJI, DMA, GNQ, ERI, FRO, FLK, GUF, PYF, GAB, GHA, GIB, GRL, GRD, GLP, GGY, GIN, GNB, GUY, IRQ, IMN, JEY, RKS, LAO, LBR, LBY, LIE, MWI, MDV, MTQ, MRT, MYT, MCO, MNE, MSR, NCL, MKD, MNP, PSE, PNG, REU, KNA, BLM, LCA, VCT, SMR, STP, SYC, SXM, SOM, SSD, SDN, SUR, TWN, TTO, TCA, VAT, VEN, SHN, TJK, ALA, SPM, COK, NIU, NFK, PRK, PLW, TUV, WLF, YEM |
| No International Travel Restriction  (44) | AIA, ATG, ARM, BES, VGB, CYM, CUW, GNQ, FLK, GUF, PYF, GIB, GLP, GGY, GNB, IMN, JEY, MDV, MTQ, MYT, MNE, MSR, NCL, MKD, MNP, REU, KNA, BLM, LCA, VCT, STP, SXM, TCA, VAT, SHN, ALA, SPM, COK, NIU, NFK, PRK, PLW, TUV, WLF |
| No SE or SNI or ITR  (82) | ABW, AFG, AIA, ALA, AND, ARM, ATG, BES, BLM, BMU, COD, COK, COM, CUW, CYM, DJI, DMA, ERI, FLK, FRO, GAB, GGY, GHA, GIB, GIN, GLP, GNB, GNQ, GRD, GRL, GUF, GUY, IMN, IRQ, JEY, KNA, LAO, LBR, LBY, LCA, LIE, MCO, MDV, MKD, MNE, MNP, MRT, MSR, MTQ, MWI, MYT, NCL, NFK, NIU, PLW, PNG, PRK, PSE, PYF, REU, RKS, SDN, SHN, SMR, SOM, SPM, SSD, STP, SUR, SXM, SYC, TCA, TJK, TTO, TUV, TWN, VAT, VCT, VEN, VGB, WLF, YEM |

| **Table S2** Workflow of missing data processing | |
| --- | --- |
| **Number of countries or regions left** | **Filter conditions** |
| 271 | The original aviation network data includes some U.S. states. |
| 228 | After merging the data for the contiguous U.S. states (excluding Alaska and Hawaii) and filtering out flight records with zero passengers. |
| 228 | With social network indicator data |
| 199 | With socioeconomic data |
| 185 | With international travel restriction data |
| 147 | With T2A data |

| **Table S3** The top 20 countries of spatial transmission potential | | | | | | | |
| --- | --- | --- | --- | --- | --- | --- | --- |
| **Index** | **ISO Code** | **Spatial Transmission Potential** | **Spatial Spreader Potential** | **Spatial Receiver Potential** | **T2A** | **SEPZ** | **Temporal**  **Infection Cluster** |
| **1** | USA | 0.115 | 0.055 | 0.061 | 13 | 3 | 1 |
| **2** | CHN | 0.111 | 0.056 | 0.055 | 0 | 2 | 1 |
| **3** | GBR | 0.105 | 0.053 | 0.052 | 31 | 1 | 1 |
| **4** | JPN | 0.087 | 0.043 | 0.044 | 16 | 2 | 1 |
| **5** | DEU | 0.083 | 0.042 | 0.042 | 27 | 1 | 1 |
| **6** | ESP | 0.078 | 0.039 | 0.039 | 31 | 1 | 1 |
| **7** | KOR | 0.073 | 0.037 | 0.037 | 20 | 2 | 1 |
| **8** | THA | 0.063 | 0.031 | 0.032 | 8 | 2 | 1 |
| **9** | ITA | 0.061 | 0.030 | 0.031 | 31 | 1 | 1 |
| **10** | FRA | 0.059 | 0.030 | 0.030 | 24 | 1 | 1 |
| **11** | CAN | 0.057 | 0.029 | 0.028 | 25 | 3 | 1 |
| **12** | MEX | 0.052 | 0.027 | 0.025 | 59 | 3 | 2 |
| **13** | HKG | 0.051 | 0.026 | 0.026 | 23 | 2 | 1 |
| **14** | ARE | 0.047 | 0.024 | 0.023 | 29 | 4 | 1 |
| **15** | NLD | 0.041 | 0.020 | 0.020 | 58 | 1 | 2 |
| **16** | SGP | 0.039 | 0.019 | 0.020 | 23 | 2 | 1 |
| **17** | CHE | 0.034 | 0.017 | 0.017 | 56 | 1 | 2 |
| **18** | VNM | 0.033 | 0.016 | 0.017 | 23 | 2 | 1 |
| **19** | IND | 0.032 | 0.016 | 0.016 | 30 | 4 | 1 |
| **20** | TUR | 0.030 | 0.015 | 0.015 | 71 | 1 | 3 |
| Spatial transmission potential: the summation of hub and authority from HITS algorithm of each country; spatial spreader potential: the hub value from HITS algorithm of each country; spatial receiver potential: the authority value from HITS algorithm of each country; T2A: the arrival date difference between the first case in the world and the first imported case of the specific country; SEPZ: spatial epidemic prevention zone based on Infomap community detection; temporal infection cluster: the groups with a similar T2A by the k-means clustering. | | | | | | | |

| **Table S4** The country lists of four spatial transmission potential levels in each spatial epidemic prevention zone | | | | |
| --- | --- | --- | --- | --- |
|  | **Q1** | **Q2** | **Q3** | **Q4** |
| **SEPZ-1** | GBR, DEU, ESP, FRA, ITA, TUR, NLD, CHE, RUS, POL, PRT, AUT, BEL, IRL, DNK, SWE, MAR | NOR, ISR, FIN, UKR, ROU, GRC, HUN, CZE, DZA, BGR, MLT, ISL | CYP, SRB, LVA, TUN, LTU, KAZ, UZB, BLR, LUX, GEO, AZE, ALB, SEN, HRV, EST, MDA, CPV, SVK | KGZ, CIV, BIH, SVN, MLI, BFA, GMB, NER, SLE |
| **SEPZ-2** | CHN, JPN, THA, KOR, SGP, HKG, MYS, AUS, IDN, VNM, PHL | NZL, KHM, MAC, MMR | GUM, BRN, FJI, MNG | VUT, TLS, TON, SLB |
| **SEPZ-3** | USA, CAN, MEX, BRA, PRI | PAN, COL, ARG, DOM, PER, CHL, CRI, CUB, JAM, SLV, BHS | ECU, GTM, HND, VIR, BRB, NIC, HTI, BLZ | URY, BOL, PRY |
| **SEPZ-4** | ARE, IND, SAU, QAT | EGY, KWT, OMN, PAK, BHR, LKA, BGD | JOR, IRN, LBN, NPL | SYR, BTN |
| **SEPZ-5** | (No country) | ZAF, ETH | KEN, NGA, MUS | TZA, UGA, AGO, RWA, ZWE, NAM, ZMB, CMR, MOZ, TGO, MDG, BWA, BEN, COG, BDI, TCD, CAF, LSO, SWZ |


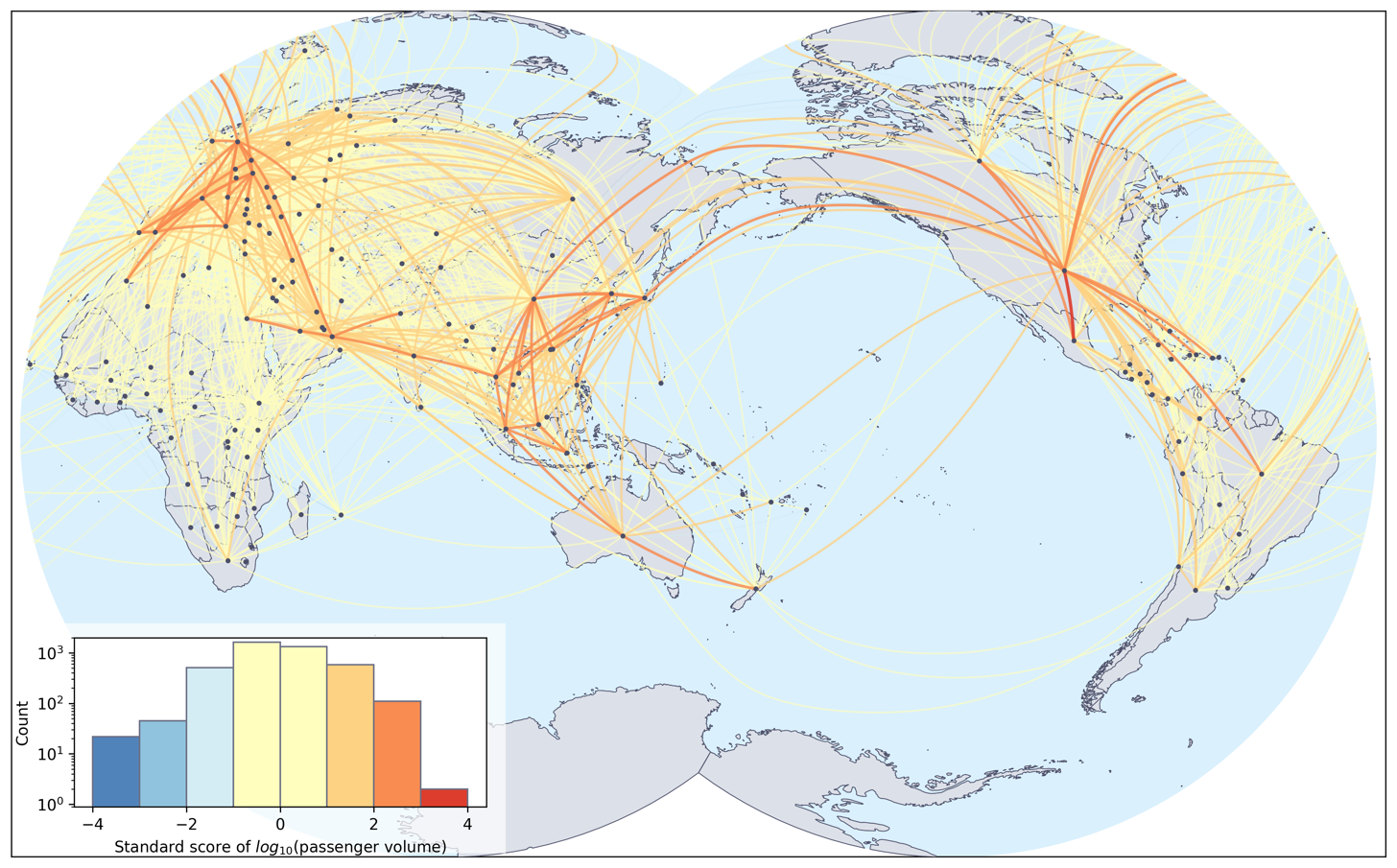


**Figure S1** The global country network. Nodes represent countries and regions, and edges represent the great circle line between countries and regions. Colors are assigned based on the standard score of the logarithmic passenger volume (see the inset for the frequency distribution), with orange and red indicating volumes greater than the mean and blue indicating volumes less than the mean.


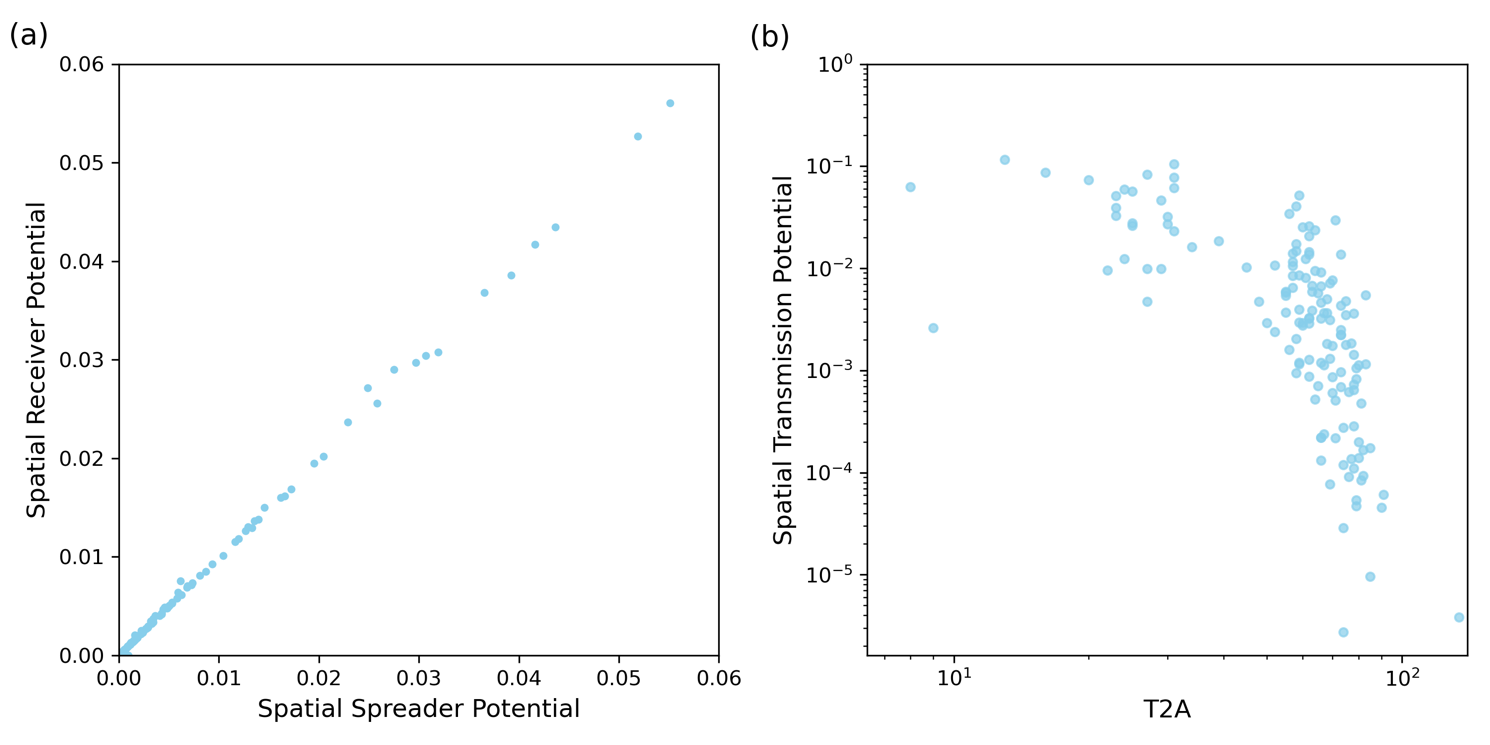


**Figure S2** The relationship between spatial spreader potential, spatial receiver potential, spatial transmission potential, and T2A. (a) Spatial spreader potential versus spatial receiver potential; (b) T2A versus spatial transmission potential
